# *IL‐4* gene polymorphisms and their relation to steroid‐induced osteonecrosis of the femoral head in Chinese population

**DOI:** 10.1002/mgg3.563

**Published:** 2019-01-29

**Authors:** Tianbo Jin, Yiming Zhang, Yao Sun, Jiamin Wu, Zichao Xiong, Zhi Yang

**Affiliations:** ^1^ Key Laboratory of Molecular Mechanism and Intervention Research for Plateau Diseases of Tibet Autonomous Region, School of Medicine Xizang Minzu University Xianyang China; ^2^ Key Laboratory of High Altitude Environment and Genes Related to Diseases of Tibet Autonomous Region, School of Medicine Xizang Minzu University Xianyang China; ^3^ Key Laboratory for Basic Life Science Research of Tibet Autonomous Region, School of Medicine Xizang Minzu University Xianyang China; ^4^ Key Laboratory of Resource Biology and Biotechnology in Western China (Northwest University), Ministry of Education Xi’an China; ^5^ Department of Orthopedics Suizhou Hospital, Hubei University Of Medicine Suizhou China; ^6^ Osteonecrosis and Joint Reconstruction Ward, Department of Joint Surgery, Hong Hui Hospital Xi’an Jiaotong University Health Science Center Xi’an China

**Keywords:** gene polymorphism, interleukin‐4 gene, steroid‐induced osteonecrosis of the femoral head, susceptibility

## Abstract

**Background:**

Steroid‐induced osteonecrosis of the femoral head (ONFH) is a debilitating disease characterized by the activation and infiltration of macrophages into the necrotic site. Interleukin‐4 (*IL‐4*) administration helped reduce the infiltration of M1 phenotypic macrophages and maintain the activation of M2 phenotypic macrophages, resulting in restriction of inflammation and decrease in osteocyte apoptosis. The aim of this study was to investigate the associations of polymorphisms of *IL‐4* gene with steroid‐induced ONFH in Chinese patients.

**Materials and methods:**

A total of 286 steroid‐induced ONFH patients and 441 healthy controls were enrolled. We evaluated 6 single nucleotide polymorphisms of the *IL‐4* gene in this case–control study.

**Results:**

We identified rs2243283 in the *IL‐4*gene was potentially associated with an increased risk of steroid‐induced ONFH in the dominant model (*p = *0.034; odds ratio [OR]: 1.40; 95% confidence intervals [CI]: 1.03–1.91) and in the log‐additive model (*p = *0.04; OR: 1.31; 95% CI: 1.01–1.71) adjusted by age and gender. Furthermore, we also observed a protective effect of rs2243289 in the dominant model (*p = *0.024; OR: 0.70; 95% CI: 0.51–0.95) adjusted by age and gender.

**Conclusion:**

These findings suggested that polymorphisms of *IL‐4* gene may be associated with susceptibility to steroid‐induced ONFH.

## 1 INTRODUCTION

Osteonecrosis of the femoral head (ONFH) is the final common pathway of a series of derangements that result in a decrease in blood flow to the femoral head leading to cellular death, fracture, and collapse of the femoral head and advanced joint destruction (Herndon & Aufranc, 1972; Moya‐Angeler, Gianakos, Villa, Ni, & Lane, 2015; Tripathy, Goyal, & Sen, 2015). In the United States, about 20,000 to 30,000 new patients are diagnosed with osteonecrosis annually, while 15,000 to 20,000 new patients are diagnosed with osteonecrosis annually in China (Jones & Hungerford, 2004; Mankin, 1992). In male patients, alcoholic was the most common aetiology (40.4%), while in female patients, idiopathic and steroid induced were the major aetiologies (38.3% and 32.9%). There were much less alcoholic female ONFH patients (5.9%) (Cui et al., 2016). Similarly, genetic factors play an important role in the development of steroid‐induced femoral head necrosis (Li et al., 2016; Wang et al., 2015; Zhang et al., 2013).

The interleukin‐4 (*IL‐4*) gene is located on the long arm of chromosome 5 (q31–33) within a cluster of other cytokine genes in human beings. *IL‐4* cytokine, a key regulator of macrophages function that downregulates CD14 levels and inhibits secretion of interferon‐gamma (IFN‐γ), *IL‐6,* and tumor necrosis factor (TNF) (Sokol, Barton, Farr, & Medzhitov, 2008), was codiscovered by Maureen Howard (Howard, 1983) and William E. Paulas well as by Ellen Vitetta and her research group in 1982. Furthermore, *IL‐4* induces differentiation of naive helper T cells (Th0 cells) to Th2 cells (Marsh et al., 1994) and apoptosis in macrophages and monocytes (Yamamoto et al., 1996). Study by Martin L. et al indicated that interleukin‐4 (*IL‐4*) could take part in the recruitment of osteoblasts and thereby be of importance in the cytokine regulation of bone resorption and healing (Lind, Deleuran, Yssel, Fink‐Eriksen, & Thestrup‐Pedersen, 1995). Thus, genetic polymorphisms in the *IL‐4* gene may affect steroid‐induced ONFH disease severity.

The above findings support the associations between genetic polymorphisms of *IL‐4* gene and the risk of steroid‐induced ONFH in different populations. However, there are not obvious evidences in the relationship of these polymorphisms with steroid‐induced ONFH. Therefore, the aim of this study was to validate the genetic association of *IL‐4* polymorphisms with the risk for steroid‐induced ONFH in Chinese population.

## 2 MATERIALS AND METHODS

### 2.1 Study population

The procedure of the study was reviewed by the Ethical Committee of Zhengzhou Traditional Chinese Medicine Traumatology Hospital, Xi'an Jiaotong University, and Xizang Minzu University and conducted in line with the Declaration of Helsinki. Written informed consent was obtained from all participants prior to their participation in the study.

A total of 286 patients with steroid‐induced ONFH (case group, 173 males and 113 females; mean age 41.43 ± 13.12 years) and 411 healthy adults (control group, 265 males and 176 females; mean age 44.60 ± 11.55 years) were consecutively enrolled between September 2014 and January 2016 at Zhengzhou Traditional Chinese Medicine Traumatology Hospital and Xi'an HongHui Hospital. All the subjects are genetically unrelated.

The diagnosis of ONFH was based on the evidence of osteonecrosis on anteroposterior and frog‐view X‐rays of both hips and/or magnetic resonance imaging (Totty et al., 1984). The Association Research Circulation Osseous (ARCO) classification system was used for radiographic evaluation (Table 1) (Gardeniers, Gosling‐Gardeniers, & Rijnen, 2014; Gardeniers,). Steroid‐induced ONFH was defined by a history of a mean daily dose ≥16.6 mg or a highest daily dose of 80 mg of prednisolone equivalent within 1 year prior to the development of symptoms or radiological diagnosis in asymptomatic cases (Du et al., 2016; Eichelbaum, Fromm, & Schwab, 2004; Zhao, Li, & Guo, 2012). Participants were excluded on the basis of having traumatic ONFH, dislocation of the hip joint, with histories of drinking more than 400 ml ethanol per week, significant familial hereditary disease, or other hip diseases. Healthy control subjects were matched with patients for gender and age (3‐year range) and recruited from subjects who did not have steroid‐induced ONFH or other related diseases on routine medical examination. Individuals with excessive use of corticosteroids, alcohol consumption, or significant familial hereditary disease were excluded.

**Table 1:** ARCO international classification of osteonecrosis

Stage	Criteria
0	Normal or nondiagnostic radiograph, bone scan, and MRI
1	A band lesion of a low signal intensity around the necrotic area is seen on MRI scans.
2	X‐ray abnormal: sclerosis, osteolysis, focal porosis
Early 3	Crescent sign. On the X‐ray and/or flattening of articular surface of femoral head.
Late 3	Collapse. On the X‐ray and/or flattening of articular surface of femoral head.
4	Osteoarthritis: joint space narrowing, acetabular changes, joint destruction

### 2.2 SNPS Selection and genotyping

All six SNPs had minor allele frequencies >5% in the HapMap Chinese Han Beijing population and were previously shown to be associated with the risk of steroid‐induced ONFH or of diseases with pathogenesis similar to that of steroid‐induced ONFH. Blood samples were collected in EDTA tubes and stored at −80°C after centrifugation at 2,000 rpm for 10 min. Genomic DNA was extracted from 2 ml whole‐blood samples using the GoldMag extraction method (GoldMag Co. Ltd, Xi'an, China) following the manufacturer's protocol. After dilution to 20 ng/μl, DNA was distributed in 96‐well plates and stored at −80°C. The DNA quantity was evaluated by spectrometry (DU 530 UV/VIS spectrophotometer, Beckman Instruments, Fullerton, CA).

The SNP genotyping was performed on the SEQUENOM MassARRAY Analyzer 4 (Agena Bioscience, Inc., San Diego, CA, USA) by Sequenom Assay Design 3.0 software (Agena Bioscience, San Diego, CA, USA) according to the manufacturer's instructions. For quality control, genotyping was performed without knowledge of the case/control status of the subjects, and a random sample of 5% of cases and controls was genotyped again by different researchers. A Sequenom MassARRAY RS1000 was used to perform the SNP genotyping.

### 2.3 Statistical analyses

The software of SPSS version 12.0 for Windows (SPSS Inc., IL, USA) and SAS 9.1 (SAS Institute, Cary, NC, USA) was used for statistical analysis. Continuous variables were expressed as mean ± SD. Statistical analysis was performed with Fisher's exact test for any 2*2 tables, Pearson's chi‐square test for non‐2*2 tables, chi‐square trend test for ordinal datum. Allele and genotype frequencies were obtained by direct counts. The genotype frequencies of each SNP among the control subjects were checked for Hardy–Weinberg equilibrium (HWE) before analysis. In the present study, all *p* values were 2‐sided, and *p* < 0.05 was considered to have statistical significance. Associations between the genotype polymorphisms and the risk of steroid‐induced ONFH were evaluated under different genetic models (codominant, dominant, recessive, and log‐additive). Odds ratios (ORs) and 95% confidence intervals (CIs) were calculated using unconditional logistic regression analysis.

## 3 RESULTS

### 3.1 Study population

Table 2 shows the general characteristics of the study population which consisted of 286 steroid‐induced ONFH patients (case group) and 411 healthy adults (control group). The mean age of the patients was 41.43 ± 13.12 years and that of the controls was 44.60 ± 11.55 years. The mean age between patients and controls was not matched in present study (*p* = 0.006), while the groups were well matched by sex (*p* = 0.915). In order to avoid the influence of these mismatch factors on our statistical results, we used them as covariates in logistic regression analysis.

**Table 2:** The comparison of age and gender between the steroid‐induced ONFH and control group

Age and gender	ONFH patients (*n* = 286)	Control (*n* = 441)	*p*
Age, year, mean ± SD	41.43 ± 13.12	44.60 ± 11.55	0.006*^,^ a
Gender, M/F	173/113	265/176	0.915^b^

Note

*p < *0.05 indicates statistical significance.

*Indicate a significant difference *p* < 0.05.

a Independent‐samples *t* test.

b Two‐sided chi‐squared test.

### 3.2 Hardy–Weinberg equilibrium and SNP alleles

The basic information about all the SNPs including gene, band, position, alleles, and HWE results are presented in Table 3. All of the six tag SNPs were in HWE among the control subjects (*p* > 0.05). The HWE test was used to check whether the surveyed population approached the genetic equilibrium and whether random sampling requirements were achieved. The differences in the frequency distribution of alleles between cases and controls were compared by Pearson chi‐square test. None of the SNPs was found to be significantly associated with the risk of steroid‐induced ONFH.

**Table 3:** Summary of the basic information on candidate SNPs examined in the *IL‐4* gene among the cases and controls, and the odds ratio estimates for steroid‐induced ONFH

SNP ID	Gene	Position	Alleles A/B	MAF		Role	HWE‐*p* a	Allele model	
				Case	Control			OR(95% CI)	*p* b
rs2243250	*IL4*	5q31.1(132009154)	C/T	0.208	0.237	Promoter	0.113	0.85(0.65–1.09)	0.197
rs2227284	*IL4*	5q31.1(132012725)	G/T	0.142	0.167	Intron	0.121	0.82(0.61–1.10)	0.193
rs2243267	*IL4*	5q31.1(132013886)	G/C	0.198	0.232	Intron	0.181	0.81(0.63–1.05)	0.116
rs2243270	*IL4*	5q31.1(132014109)	A/G	0.201	0.232	Intron	0.181	0.83(0.64–1.07)	0.158
rs2243283	*IL4*	5q31.1(132016593)	G/C	0.228	0.186	Intron	0.636	1.29(1.00–1.67)	0.054
rs2243289	*IL4*	5q31.1(132018132)	A/G	0.189	0.230	Intron(boundary)	0.346	0.78(0.60–1.01)	0.060

Note

95% CI, 95% confidence interval; A, minor alleles; B, major alleles; HWE, Hardy–Weinberg equilibrium; MAF, minor allele frequency; OR, odds ratio; SNP, single nucleotide polymorphism.

a *p* values were calculated using the exact test.

b *p* values were calculated using Pearson chi‐squared test.

### 3.3 Association between *IL‐4* and the risk of steroid‐induced ONFH

Genetic models (codominant, dominant, recessive, and log‐additive) and the genotype frequencies were used to further identify the associations between the SNPs and the risk of steroid‐induced ONFH (Table 4). The results showed that rs2243283 significantly increased the risk of steroid‐induced ONFH under the dominant model (crude OR =1.37, 95% CI: 1.01–1.86, *p* = 0.044; adjusted by age and gender OR =1.40, 95% CI: 1.03–1.91, *p* = 0.034) and log‐additive model (adjusted by age and gender OR = 1.31, 95% CI: 1.01–1.71, *p* = 0.040). However, rs2243289 showed a positive effect on steroid‐induced ONFH under the dominant model (crude OR = 0.71, 95% CI: 0.52–0.98, *p* = 0.034; adjusted by age and gender OR =0.70, 95% CI: 0.51–0.95, *p* = 0.024). There were no differences found in genotype frequencies of other SNPs between controls and patients with steroid‐induced ONFH (all *p* > 0.05, Table 4).

**Table 4:** Genotypic model analysis of the relationship between SNPs and the risk of steroid‐induced ONFH

SNP ID	Model	Genotype	Control (%)	Case (%)	Crude OR (95% CI)	*p* a	Adjusted OR (95% CI)	*p* b
rs2243283	Codominant	C/C	293 (66.6%)	169 (59.3%)	1	0.140	1	0.100
		C/G	130 (29.6%)	102 (35.8%)	1.36 (0.99–1.88)		1.39 (1.01–1.92)	
		G/G	17 (3.9%)	14 (4.9%)	1.43 (0.69–2.97)		1.48 (0.71–3.10)	
	Dominant	C/C	293 (66.6%)	169 (59.3%)	1	0.047*	1	0.034*
		C/G‐G/G	147 (33.4%)	116 (40.7%)	1.37 (1.01–1.86)		1.40 (1.03–1.91)	
	Recessive	C/C‐C/G	423 (96.1%)	271 (95.1%)	1	0.500	1	0.450
		G/G	17 (3.9%)	14 (4.9%)	1.29 (0.62–2.65)		1.32 (0.64–2.74)	
	Log‐additive	—	—	—	1.29 (0.99–1.67)	0.056	1.31 (1.01–1.71)	0.040*
rs2243289	Codominant	G/G	265 (60.1%)	194 (67.8%)	1	0.096	1	0.072
		A/G	149 (33.8%)	76 (26.6%)	0.70 (0.50–0.97)		0.68 (0.49–0.95)	
		A/A	27 (6.1%)	16 (5.6%)	0.81 (0.42–1.54)		0.78 (0.41–1.49)	
	Dominant	G/G	265 (60.1%)	194 (67.8%)	1	0.034*	1	0.024*
		A/G‐A/A	176 (39.9%)	92 (32.2%)	0.71 (0.52–0.98)		0.70 (0.51–0.95)	
	Recessive	G/G‐A/G	414 (93.9%)	270 (94.4%)	1	0.770	1	0.700
		A/A	27 (6.1%)	16 (5.6%)	0.91 (0.48–1.72)		0.88 (0.46–1.67)	
	Log‐additive	—	—	—	0.79 (0.62–1.02)	0.069	0.78 (0.60–1.00)	0.049

a *p* values were calculated by Wald test by unconditional logistic regression.

b *p* values were calculated by Wald test by unconditional logistic regression adjusted for age and gender.

* Indicate a significant difference *p* < 0.05.

### 3.4 Association of *IL‐4* haplotypes with the risk of steroid‐induced ONFH

**Figure 1:**
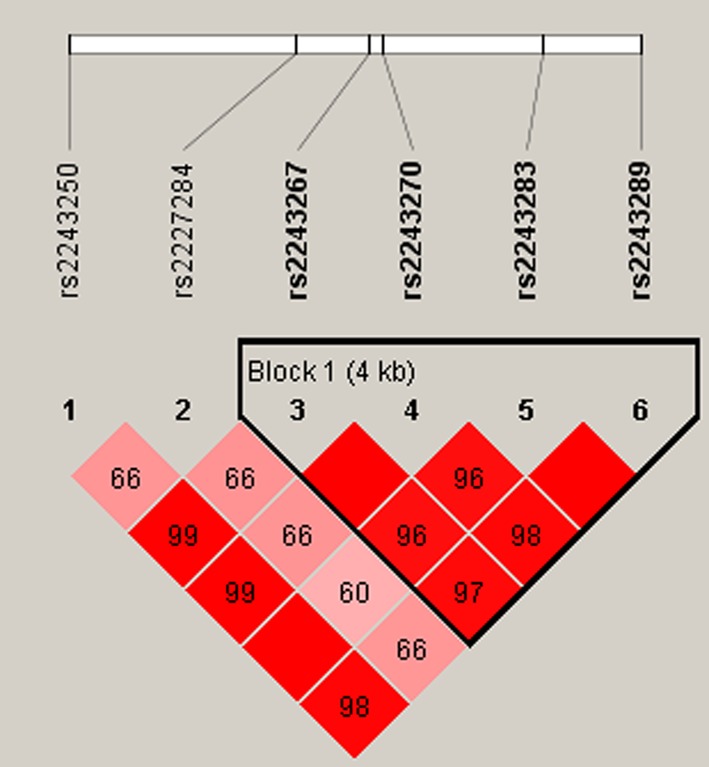
Haplotype block map for SNPs of the *IL‐4* gene. Linkage disequilibrium plots containing 6 SNPs from *IL‐4*. Red squares display statistically significant associations between a pair of SNPs, as measured by D’; darker shades of red indicate higher D’

Finally, the linkage disequilibrium and haplotype construction were detected and evaluated. One block of *IL‐4* SNPs (Figure 1) comprising rs2243267, rs2243270, rs2243283, and rs2243289 was found in studies by haplotype analysis. The results of the association between the *IL‐4* haplotype and the risk of steroid‐induced ONFH are listed in Table 5. There was no significant association between haplotype “GACA” and “CGGG” and steroid‐induced ONFH (crude *p* = 0.14, 0.16; adjusted by age and gender *p* = 0.11, 0.13).

**Table 5:** The haplotype frequencies of *IL‐4* polymorphisms and their association with the risk of steroid‐induced ONFH

Haplotype					Freq	Without adjustment		With adjusted	
	rs2243267	rs2243270	rs2243283	rs2243289		OR (95% CI)	*p* a	OR (95% CI)	*p* b
1	C	G	C	G	0.576	1	—	1	—
2	G	A	C	A	0.211	0.82 (0.63–1.07)	0.140	0.80 (0.61–1.05)	0.110
3	C	G	G	G	0.201	1.21 (0.93–1.59)	0.160	1.23 (0.94–1.62)	0.130
rare	*	*	*	*	0.012	2.98 (1.05–8.43)	0.040*	2.86 (1.01–8.11)	0.048*

a *p* values were calculated by unconditional logistic regression.

b *p* values were calculated by unconditional logistic regression adjusted for age and gender.

* Indicate a significant difference *p < *0.05.

## 4 DISCUSSION

In our case–control study, rs2243283 in the *IL‐4* gene associated with an increased risk of steroid‐induced ONFH, rs2243289 of the *IL‐4* gene was associated with decreased risk of developing steroid‐induced ONFH. Our results indicate a statistically significant difference between the steroid‐induced ONFH and control groups regarding the *IL‐4* SNPs, suggesting an association between genetic polymorphism and the susceptibility of steroid‐induced ONFH.

A pathophysiological model of steroid‐induced ONFH is a multiple hit theory and intravascular thrombosis, aberrant lipid metabolism, and steroid sensitivity have been mainly explored as a major pathogenic event (Lee et al., 2012). In this regard, candidate genes such as cytochrome P450 (*CYP3A4*) (Wang et al., 2016), insulin‐like growth factor binding proteins (*IGFBP3*) (Song et al., 2016), and adenosine triphosphate‐binding cassette B1 (*ABCB1*) (Zhang et al., 2014) have been investigated. However, until now, there has been no study concerning a gene related to cytokine. Although no study was available before for the association *IL‐4* polymorphism with steroid‐induced ONFH, this SNP has been associated with autoimmune hepatitis (Yousefi et al., 2016), osteoarthritis (Vargiolu et al., 2010), asthma (Zhang et al., 2007), chronic periodontitis (Chen, Zhang, & Wang, 2016), and other clinical features (Wadley et al., 2013). Given the repeated reports on positive associations, functional relevance did seem likely linked to the *IL‐4* polymorphism. For orthopedics, *IL‐4* can directly inhibit osteoclast precursors to differentiate into mature osteoclasts and reduce the differentiation and function of osteoclasts (Zhang et al., 2016). In addition, macrophages play an important role during the development of steroid‐induced osteonecrosis. In the previous study, Kamal et al. (2015) showed the higher number of macrophages might be a statistically relevant marker in patients who had corticosteroids as the main risk factor. *IL‐4* administration helped reduce the infiltration of M1 phenotypic macrophages and maintain the activation of M2 phenotypic macrophages (Kumar, Abbas, & Aster, 2005), resulting in restriction of inflammation and decrease in osteocyte apoptosis (Wu et al., 2016). In our study, we found polymorphisms of *IL‐4* gene are associated with the susceptibility of steroid‐induced ONFH. In present study, we found that rs2243283 of the *IL‐4* gene, which is mapped to chromosome 5q31.1, was associated with the risk of steroid‐induced ONFH in Chinese Han patients. However, rs2243289 was found to be associated with a decreased risk of steroid‐induced ONFH crude/adjusted by age and gender. Thus, the exact location and biological functions of the real causal SNPs in the *IL‐4* gene are of great interest and warrant further investigation.

There are several limitations to our study. First, the sample size (286 patients and 441 control subjects) was not large enough to generalize our results. In our study, the calculated effect sizes were relatively small for all comparisons, which may be secondary to a small sample size. But, this limitation should not be mistakenly translated into clinically insignificant results because ONFH is considered a pathophysiologically heterogeneous disease and the effect size of single gene variants could not be large. Furthermore, even though steroid is a well‐defined risk factor of nontraumatic ONFH, the subgroup of steroid‐induced ONFH had diverse underlying diseases in our study and it could affect the distributions of *IL‐4* polymorphisms. Thus, the heterogeneity among patients is a real issue in clinical practice when we are confront with steroid‐induced ONFH.

In summary, we guess that *IL‐4* rs2243283 increased the risk of steroid‐induced ONFH, and *IL‐4* rs2243289 decreased the risk of steroid‐induced ONFH. In future, we will expand our samples to verify our results. Afterward, we will do some functional studies based on the reliability results to explore how *IL‐4* gene affects the incidence of steroid‐induced ONFH.

## ETHICAL APPROVAL

All procedures performed in studies involving human participants were in accordance with the ethical standards of the Zhengzhou Traditional Chinese Medicine Traumatology Hospital, Xi'an Jiaotong University, and Xizang Minzu University and with the 1964 Helsinki declaration and its later amendments or comparable ethical standards.

## CONFLICT OF INTEREST

The authors declare no conflict of interest.

Informed consent: Informed consent was obtained from all individual participants included in the study.
